# Self-Reported Sleep Quality Using the Malay Version of the Pittsburgh Sleep Quality Index (PSQI-M) In Malaysian Adults

**DOI:** 10.3390/ijerph16234750

**Published:** 2019-11-27

**Authors:** Nor MF Farah, Teh Saw Yee, Hanif Farhan Mohd Rasdi

**Affiliations:** Program of Occupational Therapy, Faculty of Health Sciences, Universiti Kebangsaan Malaysia, 50300 Kuala Lumpur, Malaysia; sawyee_teh@hotmail.com (T.S.Y.); hanif_ot@ukm.edu.my (H.F.M.R.)

**Keywords:** psychometric properties, translation, validation, reliability, working adults

## Abstract

(1) Background: The Pittsburgh Sleep Quality Index (PSQI) is a useful tool for the assessment of subjective sleep quality in non-clinical and clinical settings. This study aimed to determine sleep quality in a general Malaysian adult population using a validated Malay version of the Pittsburgh sleep quality index (PSQI-M); (2) Methods: The original PSQI was translated into Malay following forward and backward translation guidelines. The final Malay version was administered to a sample of healthy working adults (*n* = 106; mean age: 35.3 ± 7.6 years) without history of sleep disorders. Reliability and agreement were assessed using Cronbach’s alpha, intra-class correlations coefficient (ICC), standard error of measurement (SEM), and Bland–Altman plot. Convergent validity of PSQI-M was examined with the Malay version of Epworth sleepiness scale (ESS-M) using Pearson’s correlation coefficient; (3) Results: Overall mean PSQI global score was 5.25 ± 1.85. About 45% of the sample had PSQI global score >5, indicating poor sleep quality. Total sleep duration per night was 5.95 ± 1.05 h, below the recommended amount. Sleep quality seems to be affected by age but not gender. Internal consistency as measured by Cronbach’s alpha in the whole sample was 0.74, with test–retest reliability (ICC) of 0.58 and SEM of 1.34. The PSQI test–retest scores indicated that most of the respondents (90%) lay within the 95% limits of agreement. The PSQI-M also showed significant correlation with ESS-M scores (*r* = 0.37, *p* < 0.01); (4) Conclusion: The PSQI-M showed acceptable reliability and is valid to be used in a general Malaysian adult population. Findings also indicate that a majority of the adults in our sample were experiencing inadequate sleep, thus further research is needed to identify the factors associated with poor sleep quality.

## 1. Introduction

Good sleep quality is one of the most important factors contributing to physical functioning, psychological well-being, and quality of life. Sleep quality is often used to refer to a collection of sleep measures such as total sleep duration, sleep onset latency, sleep efficiency, wakefulness after sleep onset, and daytime sleepiness [1,2]. Sleep quality can be distinguished between subjective and objective sleep quality. Shorter sleep latencies, sufficient total sleep time, reduced wake after sleep onset, and alertness during daytime are often viewed as indicators of good subjective sleep quality [3]. In addition to the measures aforementioned, good sleep quality can be objectively inferred as having proper amount and relative distribution of each of the sleep stages i.e., stage 1, stage 2, slow wave sleep, and rapid eye movement (REM), measured using polysomnography [2].

Poor sleep quality is a common issue in the modern world, with up to 36% of the adult populations having poor sleep quality in Western societies [1,2]. Poor sleep quality has been linked with adverse cardiometabolic risks, including obesity [4], diabetes [5], hypertension, and strokes [6]. Cognitive impairment [7], as well as poor health risk behaviors such as substance abuse [8], eating disorders [9], and suicide ideation [10] also appear to be associated with poor sleep quality. The understanding of importance of sleep for well-being and productivity has rapidly expanded over the last two decades, thus, the assessment of sleep quality has proven useful in a wide range of population settings in identifying those who may have undetected poor sleep quality and can help determine whether further screening and/or treatment for a sleep complaint might be warranted.

The polysomnography (PSG) has been the ‘gold standard’ method to objectively assess the quality and quantity of sleep, as well as the architecture of sleep [2]. However, this methodology has its limitations for usage in studies involving the general population, especially in areas where access to PSG is scant. In such a context, subjective assessment of sleep quality has the advantage of being relatively cheap and easy to use, which may make the method more realistic and feasible in assessing sleep quality on larger populations. Several sleep-rating measures have been developed to aid clinicians and researchers, and these questionnaires focus on subjective estimates of total sleep time, sleep maintenance, waking during the night, mood and physical feelings upon waking, and other factors that could impact sleep quality, such as comorbid conditions and medications. One of the most widely used of such questionnaires is the Pittsburgh Sleep Quality Index (PSQI) [11].

The PSQI is a 19-item, self-report questionnaire that measures subjective sleep quality over the previous month. The individual 19 items in PSQI are aggregated into seven components that assess various aspects of sleep, and the sum of these seven components yields a global score that discriminates between “good” and “poor” sleepers [11]. The questionnaire has been widely used in both non-clinical (i.e., control participants with no medical conditions) and clinical (i.e., participants reporting a medical condition) samples, and across different age groups, having been translated into and validated for different languages including French, Japanese, German, Spanish, Hebrew, Nigerian, Chinese, and Arabic [1].

In Malaysia, poor sleep quality has been reported among obese Malaysian children [12], patients with chronic periodontitis [13], tertiary students [14,15], working women [16], nurses [17], senior medical attendants [18], and night shift workers [19]. These studies had adopted various subjective assessments in determining sleep quality. Despite the increasing interest and trends in sleep quality studies in both research and clinical settings, the Malay version of PSQI has not been adequately validated and tested in a general adult population. Recently, Yunus et al. [20] validated the Malay version of PSQI in a sample of older adults who were experiencing elder abuse. However, due to the characteristics of the population sampled in the study, it may be less accurate to extend the application of the PSQI to the general adult population. Furthermore, there is a paucity of data reporting the characteristics of sleep quality among Malaysian adult population. Therefore, the present study sought to fill the noted gap in the current literature by assessing validity and reliability of the Malay version of PSQI, as well as evaluating sleep quality in a sample of Malaysian adults.

## 2. Materials and Methods

### 2.1. Translation Procedures

Permission to translate the PSQI into Malay was obtained from Mapi Research Trust, the official distributor for PSQI. Translation was performed according to the World Health Organization (WHO) process of translation and adaptation of instruments, which included forward and backward translations. Forward translation was the translation of the English version of PSQI into Malay language by two sleep researchers who were proficient in both English and Malay. Face validity was performed by two independent researchers in health sciences who were proficient in both languages. They were asked to identify and resolve the inadequate expressions or concepts of the translation, as well as any discrepancies between the forward translation and the original English version of PSQI. An independent translator whom had no knowledge of the assessment then performed the backward translation process. Comparison between the original English and the backward translated English versions were then carried out and any discrepancies were discussed before any final adjustments were made to the questionnaire.

### 2.2. Pilot Study

In order to assess the clarity and appropriateness of expressions of the items of the translated questionnaire, the PSQI-M (Pittsburgh Sleep Quality Index) was pre-tested in a pilot study of ten individuals. Ten adults with no history of sleeping problems were recruited for this purpose in which they completed the PSQI-M, twice within the period of 2 to 4 weeks. After completion of the assessment, they were asked about any words they did not understand as well as any words or expression that they found ambiguous. The final version of PSQI-M was finalized following minor suggestions obtained from the pilot study. The internal consistency of the translated PSQI-M in the pilot study, measured by Cronbach’s α coefficient, was 0.66.

### 2.3. Participants

This cross-sectional study was conducted among adults working in a university satellite campus in Kuala Lumpur. Those aged between 21 to 60 years old were approached to participate in the study. Individuals who worked on shifts, pregnant, using sleep medications, or clinically diagnosed with idiopathic insomnia, depression, obstructive sleep apnea, or other sleep disorders were excluded. An adapted insomnia checklist was used to exclude respondents who had symptoms of insomnia [21]. After the screening and selection process, a total of 106 adults met the inclusion criteria and agreed to participate in the study. After detailed explanation, written and oral informed consent was obtained from all individual respondents. Malay was the native language for all respondents. All respondents were approached in person by the investigator to complete two questionnaires: (i) PSQI-M to assess sleep quality, and (ii) Epworth sleepiness scale Malay version (ESS-M) to assess daytime sleepiness. All respondents received the PSQI-M twice and were informed about the forthcoming retest, which would take place within 2–4 weeks after the first test. This study was approved by the Research and Ethics Committee of University Kebangsaan Malaysia Medical Centre (UKM PPI/111/8/JEP-2017-738).

### 2.4. Pittsburgh Sleep Quality Index Malay Version (PSQI-M)

PSQI is a standardized, self-administered questionnaire developed by Daniel J. Buysse that evaluates retrospective sleep quality and disturbances within the past month [11]. The translated PSQI-M consists of the same items as the original instrument; it comprises 19 items forming seven subscales: (1) sleep quality (1 item), (2) sleep latency (2 items), (3) sleep duration (1 item), (4) sleep efficiency (3 items), (5) sleep disturbance (9 items), (6) sleep medication (1 item), and (7) daily dysfunction (2 items). The PSQI-M was evaluated following the original scoring system. Each component has a score that ranges from 0 to 3. The scores of seven components will be summed to yield a PSQI global score ranging from 0 to 21. Respondents with a global score of greater than 5 are classified as ‘poor sleepers’, while those with a score of 5 or less are classified as ‘good sleepers’. The original PSQI had an internal consistency of 0.83 [11].

### 2.5. Epworth Sleepiness Scale Malay Version (ESS-M)

Epworth Sleepiness Scale (ESS) is an eight-item, self-report measure of daytime sleepiness [22]. Respondents indicate, on a four-point Likert-type scale (0 = never, 3 = high chance), the likelihood that they will ‘doze off or fall asleep’ in eight different conditions, such as riding as a passenger in a car. The responses are summed to yield a total score ranged from 0 to 24, with higher scores indicating greater sleepiness during common daily activities. ESS total scores of >10 have been proposed to indicate excessive daytime sleepiness [23]. The ESS has been previously translated into Malay and tested for internal consistency (Cronbach’s *α* = 0.73) and test–retest reliability (ICC = 0.60).

### 2.6. Statistical Analyses

All statistics were performed using IBM SPSS Statistics for Windows, version 20 (IBM Corp., Armonk, NY, USA). Descriptive analysis of sleep quality was presented as mean + standard deviation (SD) for continuous or n (%) for categorical variables. Subgroup differences were tested with t-test and Mann–Whitney U-test, whichever was relevant. Kruskal–Wallis one-way analysis of variance was used to test subgroup PSQI differences. *p* values less than 0.05 were considered significant. Cronbach’s alpha values were computed to determine the internal consistency of PSQI-M. Test–retest reliability was evaluated using intra-class correlation coefficient (ICC). Measurement error was assessed with differences between test and retest plotted against the means of the two measurements by Bland–Altman plots with 95% CI and 95% limits of agreement (LOA). The measurement errors reflect the within intra-individual variation and were estimated as the standard error of the measurement (SEM) [24]. The convergent validity of PSQI-M was examined using ESS-M to explore the association between these two measures using Pearson’s correlation coefficient.

## 3. Results

### 3.1. Internal Consistency of PSQI-M

To assess the PSQI internal consistency, Cronbach’s-α coefficients were computed for the whole sample. The PSQI-M showed internal consistency values of 0.74 (test) and 0.68 (retest), indicating acceptable internal consistency. There was no improvement in internal consistency for PSQI-M when any of the items were deleted.

### 3.2. Test Retest Reliability

ICC was used to examine the reliability between test and retest, as presented in Table 1. The ICC for PSQI-M global score was 0.58 (95% CI 0.43–0.69), indicating a fair test–retest reliability. The ICCs for PSQI-M subcomponents ranged from 0.40 (95% CI 0.23–0.55) for daily dysfunction to 0.62 (95% CI 0.50–0.73) for sleep duration. Standard error of measure (SEM) for PSQI global score was 1.34, which is considered acceptable in this study. Sleep medication (component 6) was excluded when examining test–retest reliability because those who were on sleep medications were excluded in this study, thus sleep medication had zero variance.

### 3.3. Bland–Altman Plot of the Mean PSQI Global Score

**Figure 1:**
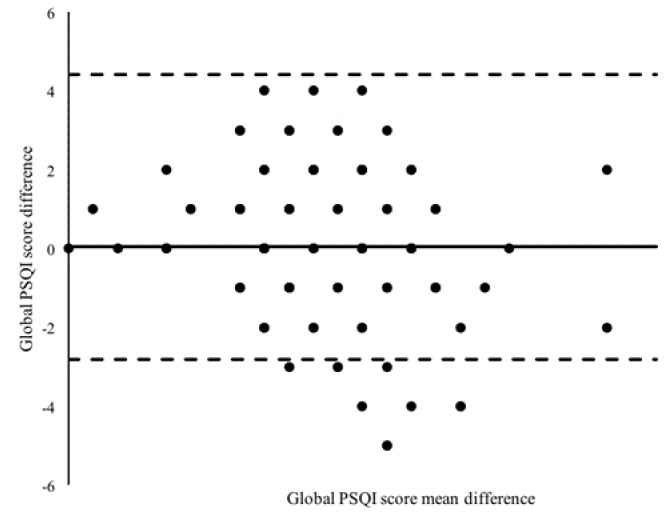
Bland–Altman dispersion plot of the mean PSQI global score (*n* = 106). PSQI: Pittsburgh Sleep Quality Index.

Figure 1 shows the analysis of absolute agreement of the PSQI global score with all subcomponents and after excluding the component on sleep medication. The mean of global PSQI score was close to 0, indicating good reliability. In total, 96 respondents out of 106 (90%) lay within the 95% limits of agreement.

### 3.4. Convergent Validity

Table 2 shows the convergent validity for PSQI-M with ESS-M. In the sample, PSQI global score correlated significantly with ESS-M total score (*p* < 0.001). In addition, the ESS-M total score correlated significantly with sleep disturbance (r = 0.35, *p* = 0.002) and daytime dysfunction components of the PSQI-M (r = 0.48, *p* = 0.007). Component 6 (sleep medication) was excluded from analysis.

### 3.5. Subject Characteristics, Sleep Quality, and Daytime Sleepiness

**Figure 2:**
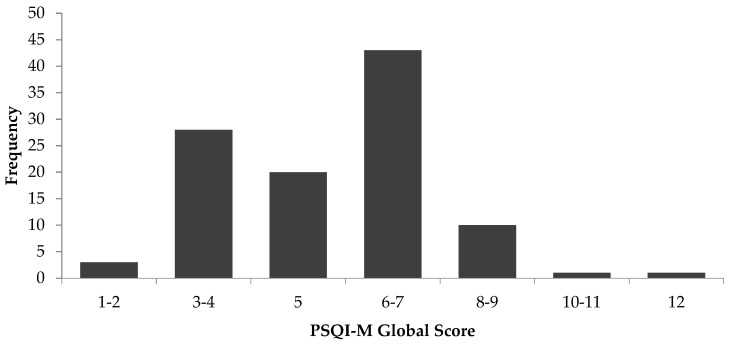
Frequency distribution of PSQI-M global score (*n* = 106). PSQI: Pittsburgh Sleep Quality Index.

A total of 106 respondents completed the study. The descriptive characteristics and scores for PSQI-M (sleep quality) and ESS-M (daytime sleepiness) of respondents are presented in Table 3. Most of the respondents (95%) were of Malay ethnicity. Fifty-seven (54%) of them were females and 49 (46%) were males. The mean age of the respondents was 35.3 ± 7.6 years. The PSQI-M global score of the sample ranged from 1 to 12, as shown in Figure 2. The mean global PSQI score for the total sample was 5.25 ± 1.85. Overall, 45% of the sample had a global PSQI score >5, indicating a disturbance in sleep quality. The reported total sleep duration per night was 5.9 ± 1.0 h. Only 24.5% of the sample obtained the recommended amount of sleep at night, which is between 7–9 h. Between gender, women appeared to have a higher PSQI global score (5.6 ± 1.9) and lower total sleep duration (5.8 ± 1.0 h) compared to men (4.9 ± 1.7; 6.0 ± 1.0 h.), though these differences were not significant. The mean total score for ESS-M was 7.3 ± 3.7. About 22% had an ESS total score >10, indicating excessive daytime sleepiness. No differences were observed for daytime sleepiness between men (7.4 ± 3.5) and women (7.1 ± 3.9). 

### 3.6. Sleep Quality and Daytime Sleepiness According to Age Groups

Table 4 shows the comparison of PSQI-M and ESS-M scores between two age groups, i.e., young adults (18–39 years) and middle-aged adults (40–60 years). The PSQI-M global scores seemed lower in young adults (4.4 ± 2.2) compared to middle-aged adults (5.5 ± 2.2), though the difference was not significant. However, the young adults reported significantly longer sleep duration (6.3 ± 0.8 h) compared to middle-aged adults (5.8 ± 1.0 h). They also demonstrated significantly lower sleep disturbance and daytime sleepiness compared to middle-aged adults. 

## 4. Discussion

In the present study, we determined the internal consistency, reliability, and convergent validity of the PSQI Malay version and tested it in a sample of Malaysian adults without sleeping disorders. Although PSQI is routinely used in research and clinical applications in various age groups, the reliability and validity of the Malay version had not been established in this particular population. Our findings indicate that the PSQI-M is appropriate for use in a healthy adult population.

The PSQI-M showed acceptable internal consistency, with Cronbach’s alpha of 0.74. Our findings were corroborated by other PSQI validation studies in different languages, i.e., Cronbach’s alpha for PSQI-Greek was 0.76 [25], PSQI-Persian 0.77 [26], PSQI-Portuguese 0.70 [27], and PSQI-Arabic 0.65 [28]. The test–retest method employed to assess the reliability of PSQI-M yielded an ICC of 0.58. This value was lower than that reported by the Portuguese version (ICC 0.76) [27], Greek version (ICC 0.82) [25], and the Thai version (ICC 0.89) [29]. However, the SEM value indicated that only 6% of error may have occurred in the test–retest period. We also utilized the Bland–Altman plot to assess agreement between the differences and averages of scores on the test–retest [30]. The plot showed that most respondents lay within the 95% limits of agreement and only 10 out of 106 respondents were beyond the limits. It is plausible that a change in sleeping habits had occurred in these respondents during the test–retest period and this might have affected the ICC and measurement error estimates. Our finding also demonstrated an adequate convergent validity with the ESS-M. The PSQI global score significantly correlated with ESS-M, which indicates an individual who has poor sleep quality is very likely to display daytime sleepiness. The correlation coefficient obtained in this study was greater than that reported by studies using PSQI-Greek (*r* = 0.30) [25], PSQI-Persian (*r* = 0.26) [26], and the original English PSQI (*r* = 0.11) [31]. The daytime dysfunction component in PSQI correlated best with ESS total scores, indicating that daytime sleepiness may affect daytime dysfunction in those who had poor sleep quality. 

With regards to sleep quality, the present study showed that about 45% the adults in the sample had poor sleep quality, represented by PSQI global score >5. The average total sleep duration at night was 5.9 ± 1.0 h, below the recommended amount of between 7–9 h [32]. Our finding seems to corroborate that of Singh et al. [13] which reported that the prevalence of poor sleep quality in a sample of healthy Malaysian adult controls (mean age 31.5 ± 9.7 years) was 48%. Jurado-Fasoli et al. [33] in their recent study reported that the PSQI global score in a sample of sedentary, middle-aged Spanish adults (mean age 53.7 ± 5.1 years) was 5.6 ± 3.5 with a total sleep duration of 5.9 ± 0.8 h, a finding that was very similar to ours. Meanwhile, a study among Chinese civil servants showed a global PSQI score of 4.6 ± 2.4 and total sleep duration of 7.9 ± 1.0 h [34], indicating better sleep quality in Chinese compared to Malaysian adults. Sleep quality and total sleep duration were not different between men and women in the present study, and this finding is supported by Mollayeva et al. [1], who reported a lack of consistency in gender differences with regards to sleep quality. Our study also showed that middle-aged adults were characterized with a lower total sleep duration, greater sleep disturbances (defined as factors affecting the quality of sleep), and daytime sleepiness compared to young adults. In support of our findings, Gadie et al. [6] had reported that sleep quality generally decreases across the lifespan and considerably after the age of 50, and are more likely to display inefficient sleeping, characterized by long periods spent in bed while not asleep. Evidence from prospective studies have identified female gender, depressed mood, and physical illness as general risk factors for developing sleep disturbances in later life [35]. Psychosocial factors such as greater work responsibilities, child rearing, and family affairs can also affect sleep quality to a greater extend in middle-aged adults compared to young adults. Aazami et al. [16] in their study had reported that sleep quality among Malaysian working women between 30–50 years was disturbed by work-family conflict, i.e., struggling for the amount of time needed to be spent with family as well as high levels of strain in the workplace.

There are potential limitations to this study. Firstly, this study did not include objective measurements of sleep duration, for example sleep actigraphy, therefore the reported total sleep duration in this study can be subjected to reporting biases. We also did not conduct a structured clinical interview or assessment to detect the presence of a clinical sleep disorder during subject selection process and only relied on past medical history to provide an approximation of subjects who may or may not have current sleep disorders. Secondly, our study was conducted on a relatively homogenous population, consisting of civil servants from a single workplace. While our respondents had somewhat different job backgrounds, i.e., lecturers, administration staff, and clinicians (i.e., optometrists, physiotherapists, dental assistants), working in a health science campus meant they were exposed to the same physical work environment, work schedules, and culture. Studies have shown that psychosocial work environment and work scheduling [36], as well as types of occupations [34,37], have an impact on sleep quality, therefore the generalizability of these findings may be limited to similar working environments and job backgrounds. Further studies may be needed to evaluate sleep quality among blue-collared workers and those working in private organizations. Thirdly, as this was a cross-sectional study, our sample size was relatively small and only 10 participants were recruited for the pilot study. However, this number was approximately 10% of the parent study and considered to be sufficient, as suggested by Connelly [38], while Hill [39] recommended the size of between 10–30 participants when conducting pilots for survey-based research. The internal consistency for our pilot study (α = 0.66) was lower than the parent study (α = 0.74). As the main purpose of the pilot study was to pre-test the internal consistency of the PSQI-M prior to the commencement of the main study, an alpha value of between 0.64–0.85 was considered to be adequate [40]. According to Yurdugül [41], the accuracy of the alpha would improve with a larger sample size. Notably, the alpha value obtained from the parent study yielded a greater value compared to that of the pilot sample. In addition, our sample size was not evenly distributed between the two adult age groups. As our primary aim was to validate the PSQI-M and gender comparison of sleep quality was not premeditated, the task of ensuring equal sample distribution for both genders was overlooked. Finally, because we excluded participants who were on sleep medications, the internal consistency, test–retest reliability, and convergent validity related to component 6 of the PSQI-M was not determined in this study.

## 5. Conclusions

In summary, this study provides evidence to support the use of the PSQI-M in a general Malaysian adult population. In addition, our findings also indicate that the working adults in our sample are not getting sufficient sleep at night and that sleep quality is lower in middle-aged adults compared to young adults. Further studies are warranted to identify the variables contributing to compromised sleep quality in this population.

## Figures and Tables

**Table 1:** Test–retest reliability for PSQI-M global score and subcomponents.

PSQI Subcomponents		Test M ± SD	Retest M ± SD	ICC ^a^	95% CI	95% LoA	SEM
PSQI Global Score		5.30 ± 1.85	5.36 ± 2.47	0.58	0.43–0.69	−2.81–4.41	1.34
C1	Sleep Quality	0.89 ± 0.59	0.82 ± 0.60	0.59	0.45–0.70	0.08–0.50	0.38
C2	Sleep Latency	0.72 ± 0.71	0.84 ± 0.76	0.54	0.40–0.66	−0.42–0.56	0.50
C3	Sleep Duration	1.35 ± 0.72	1.36 ± 0.78	0.62	0.50–0.73	0.45–1.31	0.46
C4	Sleep Efficiency	0.49 ± 0.76	0.44 ± 0.81	0.53	0.38–0.65	−0.05–1.10	0.54
C5	Sleep Disturbance	1.19 ± 0.50	1.13 ± 0.48	0.45	0.29–0.59	0.00–0.52	0.36
C7	Daytime Dysfunction	0.67 ± 0.53	0.66 ± 0.63	0.40	0.23–0.55	0.47–1.29	0.45

PSQI: Pittsburgh Sleep Quality Index, M: Mean, SD: Standard deviation, ICC: intraclass correlation coefficient, CI: Confidence interval, LoA: Limits of agreement, SEM: Standard error measure. ^a^ Two-way mixed model, absolute agreement, and single measure were used.

**Table 2:** Correlation coefficient, *r* between PSQI-M and ESS-M scores.

PSQI Subcomponents	*r*	*p*
PSQI Global score	0.37	<0.001
C1 Sleep Quality	0.18	0.345
C2 Sleep Latency	0.13	0.385
C3 Sleep Duration	0.15	0.329
C4 Sleep Efficiency	0.06	0.463
C5 Sleep Disturbance	0.35	0.002
C7 Daytime Dysfunction	0.48	0.007

PSQI: Pittsburgh Sleep Quality Index, ESS: Epworth Sleepiness Scale.

**Table 3:** Characteristics of subjects, sleep quality, and daytime sleepiness (*n* = 106).

Characteristics	*n* (%)	Mean ± SD	Minimum	Maximum
**Gender**				
Male	049 (46)			
Female	057 (54)			
**Race**				
Malay	101 (95)			
Chinese	003 (3)			
Indian	002 (2)			
**Age (years)**		35.3 ± 7.6	21.0	57.0
Young Adult (21–39 years)	083 (78)	32.4 ± 4.3	21.0	39.0
Middle-Aged (40–60 years)	023 (22)	48.5 ± 4.0	40.0	57.0
**PSQI Global Score**		5.25 ± 1.85	1	12
(C1) Sleep Quality		0.89 ± 0.59	0	3
(C2) Sleep Latency		0.72 ± 0.71	0	3
(C3) Sleep Duration		1.87 ± 0.61	0	3
(C4) Sleep Efficiency		0.49 ± 0.75	0	3
(C5) Sleep Disturbance		1.19 ± 0.50	0	3
(C6) Sleep Medication		0.00 ± 0.00	0	3
(C7) Daytime Dysfunction		0.67 ± 0.53	0	2
Total Sleep Duration (hour)		5.95 ± 1.05	4	9
**ESS Total Score**		7.26 ± 3.73	0	19

PSQI: Pittsburgh sleep quality index, ESS: Epworth sleepiness scale, SD: Standard deviation.

**Table 4:** Comparison of PSQI-M and ESS-M scores according to age.

Domains	Mean ± SD		*p* Value	Cohen’s d
	Young Adult (21–39 years)	Middle-Aged (40–60 years)		
Global PSQI Score	4.4 ± 2.2	5.5 ± 2.2	0.06	−0.18
(C1) Sleep Quality	0.7 ± 0.6	0.9 ± 0.6	0.13	−0.15
(C2) Sleep Latency	1.0 ± 1.0	0.8 ± 0.7	0.36	−0.09
(C3) Sleep Duration	0.9 ± 0.5	1.5 ± 0.8	<0.001	−0.33
(C4) Sleep Efficiency	0.4 ± 0.8	0.5 ± 0.8	0.52	−0.06
(C5) Sleep Disturbance	1.0 ± 0.4	1.2 ± 0.5	0.04	−0.20
(C7) Daytime Dysfunction	0.5± 0.5	0.7 ± 0.7	0.31	−0.10
ESS Overall Score	5.6 ± 3.0	7.7 ± 3.8	0.02	−0.22

PSQI: Pittsburgh sleep quality index, ESS: Epworth sleepiness scale, SD: Standard deviation.
